# Genome Sequences of Chikungunya Virus Isolates from Bolivia

**DOI:** 10.1128/MRA.00230-20

**Published:** 2020-04-16

**Authors:** Caio M. B. França, Roxana Loayza, Yelin Roca, Ana Maria Montaño Arias, Freddy Tinajeros, Jose R. Loaiza, Anshule Takyar, Robert H. Gilman, Matthew J. Miller

**Affiliations:** a Department of Biology, Southern Nazarene University, Bethany, Oklahoma, USA; b U.F. Biología Molecular, Centro Nacional de Enfermedades Tropicales (CENETROP), Santa Cruz, Bolivia; c Asociación Benéfica PRISMA, Santa Cruz, Bolivia; d Instituto de Investigaciones Científicas y Servicios de Alta Tecnología (INDICASAT AIP), City of Knowledge, Panama City, Republic of Panama; e Smithsonian Tropical Research Institute, Balboa, Republic of Panama; f Programa Centroamericano de Maestría en Entomología, Universidad de Panamá, Panama City, Republic of Panama; g Department of Public Health and Department of Microbiology, University of Oklahoma, Norman, Oklahoma, USA; h Bloomberg School of Public Health, John Hopkins University, Baltimore, Maryland, USA; i Sam Noble Museum of Natural History, University of Oklahoma, Norman, Oklahoma, USA; DOE Joint Genome Institute

## Abstract

We generated nine coding-complete chikungunya virus genome sequences from blood samples collected during the early 2015 outbreak in Bolivia. Relative to other publicly available chikungunya sequences, the Bolivian samples represent a monophyletic group, suggesting that a single lineage was widely circulating in the country between February and May 2015.

## ANNOUNCEMENT

**FIG 1:**
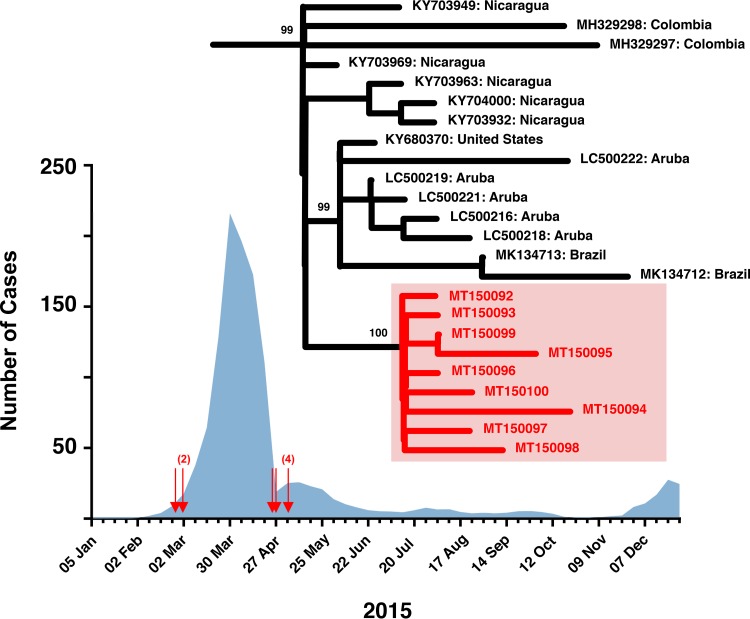
Four-week moving average of chikungunya case count across Bolivia. Red arrows indicate the collection dates for samples sequenced in this study. Maximum likelihood phylogeny was pruned to the clade, including the nine samples. Three nodes have significant approximate bootstrap support (>90).

Among the Andean nations of South America, Bolivia has had the highest incidence of chikungunya and postinfection chronic disease (1). In Bolivia, chikungunya virus was first detected in early 2015, with cases of disease peaking between March and May 2015 (Fig. 1). Here, we report nine chikungunya (*Togaviridae*: *Alphavirus*) genome sequences for isolates from Bolivia.

Febrile patients were screened for chikungunya virus at the Cenetrop national tropical medicine laboratory. We selected nine archived samples (maximum of 1 passage) for sequencing; samples were selected at random (Table 1). All isolates came from blood-extracted RNA (QIAamp viral RNA minikit; Qiagen) with unambiguously positive quantitative PCR (qPCR) tests (Pan American Health Organization [PAHO] diagnostic kits). Seven of the nine samples were from Santa Cruz de la Sierra. We also included one sample from Cochabamba and one sample from Trinidad. We generated cDNA using random hexamers via reverse transcriptase PCR (RT-PCR) (TaqMan reverse transcription reagents; Applied Biosystems). We amplified the chikungunya genome using a multiplex tiled amplicon approach (2). All samples were pooled and sequenced on a single Oxford Nanopore MinION R9.4 flow cell, generating 2,776,384 reads.

**TABLE 1:** Results of sequencing efforts for nine chikungunya virus isolates from Bolivia

Sample name	Collection date	City, department	No. of uncorrected reads	No. of corrected and trimmed reads/% mapped	Avg depth of coverage (×)	GenBank accession no.
639-15	24 Feb 2015	Santa Cruz de la Sierra, Santa Cruz	438,120	98,367/100	3,834	MT150092
4866-15	1 Mar 2015	Santa Cruz de la Sierra, Santa Cruz	495,057	110,170/>99.9	4,055	MT150093
4990-15	1 Mar 2015	Santa Cruz de la Sierra, Santa Cruz	369,957	69,285/100	2,590	MT150094
5037-15	26 Apr 2015	Cochabamba, Cochabamba	307,502	61,182/100	2,145	MT150095
5041-15	4 May 2015	Santa Cruz de la Sierra, Santa Cruz	243,726	34,567/100	1,342	MT150096
5046-15	4 May 2015	Santa Cruz de la Sierra, Santa Cruz	281,700	53,600/100	2,165	MT150097
5038-15	4 May 2015	Santa Cruz de la Sierra, Santa Cruz	80,384	28,428/>99.9	922	MT150098
746-15	4 May 2015	Santa Cruz de la Sierra, Santa Cruz	163,681	12,256/100	525	MT150099
710-15	27 Apr 2015	Trinidad, Beni	128,959	4,657/>99.9	193	MT150100

Base calling was done in real time using Albacore v2.3.1, which implements quality filtering (QC), using only QC-passed reads in subsequent analyses. We demultiplexed and trimmed adapters and barcodes using qcat v1.1.0 (https://github.com/nanoporetech/qcat), which detected barcodes in 2,538,578 reads (>91%) and assigned only 80 out of 2,538,578 (<0.0004%) reads to barcodes BC10 to BC12 (not used in this study, but assignable in the qcat demultiplexing algorithm). This suggests negligible read misassignment during demultiplexing. The average read length for QC-passed reads was 325.4 bp (range, 100 to 3,727 bp).

For our highest read count sample (4866-15), we error corrected, trimmed, and *de novo* assembled reads in Canu v1.9 (3). The resulting assembly was fragmented, so we selected the largest contig to identify the closest whole chikungunya genome on GenBank using BLAST (2 March 2020) (4) to guide reference-based assembly. The BLAST search returned GenBank accession number KY703969.1 as the closest match. We used Canu to correct and trim all nine samples. After trimming, we retained 836,301 reads with an average read length of 345.9 base pairs (range, 184 to 496 base pairs). We mapped these reads to the sequence of KY703969.1 using Minimap2 (5) implemented in Geneious v2020.0.5 (6). For each sample, we generated a consensus sequence. All nine consensus sequences along with that from KY703969.1 were aligned in Geneious, and we visually corrected homoplasy indel errors, which are common to Oxford Nanopore-derived sequences (7). Default parameters were used for all bioinformatic tools, unless otherwise specified. The final sequence length for all nine genomes is 11,182 nucleotides (because each sequence was generated from homologous amplicons tiled across the coding region, they have the same start and endpoint), representing 99.6% of the nonstructural and structural coding regions. One sample (710-15) has uncalled bases due to poor coverage in the structural protein-coding region (163 nucleotides; 1.5%); all remaining sequences have no ambiguous bases. The average G+C content for all nine sequences is 50.7% (range, 50.5% to 50.7%).

We downloaded from GenBank the top 400 BLAST hits to sample 4866-15 (2 March 2020) and filtered out sequences without a month and year of sample collection. We aligned remaining sequences with our nine sequences using MAFFT (8), as implemented in Geneious, and trimmed the alignment to the coding region recovered in our sequences. We generated a maximum likelihood phylogeny using IQ-Tree v2.0-rc1 (9). We found that the nine Bolivian sequences are part of the widespread Asian-Caribbean chikungunya genotype and form a unique clade that was part of a larger monophyletic lineage primarily containing sequences from Nicaragua, Aruba, Colombia, and the United States (Fig. 1). The monophyly of our nine samples supports the hypothesis that a single lineage was widely circulating in Bolivia during the early 2015 chikungunya outbreak.

### Data availability.

Genome sequences are available in GenBank under accession numbers MT150092 to MT150100. Sequencing reads are available in the SRA database under BioProject accession number PRJNA609363. The input, output, and complete maximum likelihood phylogenetic tree are available at https://doi.org/10.6084/m9.figshare.11938047.
